# Attention-Modulated Cortical Responses as a Biomarker for Tinnitus †

**DOI:** 10.3390/brainsci14050421

**Published:** 2024-04-25

**Authors:** Matthew L. Richardson, Jiaxin Luo, Fan-Gang Zeng

**Affiliations:** 1 Department of Otolaryngology—Head and Neck Surgery, University of California at Irvine, Irvine, CA 92697, USA; fzeng@hs.uci.edu; 2 Center for Hearing Research, University of California at Irvine, Irvine, CA 92697, USA; 3 Department of Biomedical Engineering, University of California at Irvine, Irvine, CA 92697, USA; 4 Departments of Anatomy and Neurobiology, Cognitive Sciences, University of California at Irvine, Irvine, CA 92697, USA

**Keywords:** tinnitus, evoked potentials, attention, biomarker, human

## Abstract

Attention plays an important role in not only the awareness and perception of tinnitus but also its interactions with external sounds. Recent evidence suggests that attention is heightened in the tinnitus brain, likely as a result of relatively local cortical changes specific to deafferentation sites or global changes that help maintain normal cognitive capabilities in individuals with hearing loss. However, most electrophysiological studies have used passive listening paradigms to probe the tinnitus brain and produced mixed results in terms of finding a distinctive biomarker for tinnitus. Here, we designed a selective attention task, in which human adults attended to one of two interleaved tonal (500 Hz and 5 kHz) sequences. In total, 16 tinnitus (5 females) and 13 age- and hearing-matched control (8 females) subjects participated in the study, with the tinnitus subjects matching the tinnitus pitch to 5.4 kHz (range = 1.9–10.8 kHz). Cortical responses were recorded in both passive and attentive listening conditions, producing no differences in P1, N1, and P2 between the tinnitus and control subjects under any conditions. However, a different pattern of results emerged when the difference was examined between the attended and unattended responses. This attention-modulated cortical response was significantly greater in the tinnitus than control subjects: 3.9-times greater for N1 at 5 kHz (95% CI: 2.9 to 5.0, *p* = 0.007, η_p_^2^ = 0.24) and 3.0 for P2 at 500 Hz (95% CI: 1.9 to 4.5, *p *= 0.026, η_p_^2^ = 0.17). We interpreted the greater N1 modulation as local neural changes specific to the tinnitus frequency and the greater P2 as global changes to hearing loss. These two cortical measures were used to differentiate between the tinnitus and control subjects, producing 83.3% sensitivity and 76.9% specificity (AUC = 0.81, *p *= 0.006). These results suggest that the tinnitus brain is more plastic than that of the matched non-tinnitus controls and that the attention-modulated cortical response can be developed as a clinically meaningful biomarker for tinnitus.

## 1. Introduction

Tinnitus is the phantom perception of sound without an external acoustic source. This auditory disorder affects 10–15% of the general population, including 1–2% who suffer debilitating symptoms that require medical attention (e.g., [1]). Because the physiological mechanisms underlying tinnitus are not clear, there are several management protocols to alleviate tinnitus symptoms, but there is no cure at present. Furthermore, the identification and diagnosis of tinnitus rely mostly on self-reports without any objective biomarkers for its presence or severity.

Analogous to phantom limb pain, tinnitus has been traditionally hypothesized to involve cortical reorganization following peripheral deafferentation due to aging, noise exposure, or other factors [2,3,4,5,6]. At the systems level, this cortical reorganization can be modelled as increased central gain, noise, or variance [6,7,8,9]. Attempts have been made to test these hypotheses but achieved limited success, especially in applying these models to clinical diagnosis and treatment of tinnitus in human patients (e.g., [10,11,12]).

Several lines of evidence have indicated that attention plays an important role in tinnitus perception and its interactions with external sounds. Because deafferentation does not always produce phantom percepts, attention may serve as a gate to control the conscious awareness and processing of tinnitus [4,13,14,15,16]. Moreover, attention can change central gain or noise, potentially modulating tinnitus loudness, quality, and annoyance [9,13,15,17]. Mediated by attention, tinnitus may serve either as a competing internal signal to reduce the capacity for processing external sounds [18,19,20,21] or as a compensating internal signal to maintain normal sensory or cognitive processing following deafferentation [22,23].

Most electrophysiological studies of tinnitus, however, have used a passive, no-task paradigm and produced inconsistent findings in differentiating between the tinnitus and non-tinnitus brain [6,7,24,25,26,27,28,29,30,31]. There were a few exceptions that used attention as the main factor to differentiate neural responses between tinnitus and control subjects. For example, Jacobson et al. (1996) showed not only greater cortical responses (N1) to an attended 500-Hz or 1-kHz tone than the same tone unattended, but more importantly that this attention-modulated response was greater in tinnitus than audiological-normal control subjects [32]. Additionally, Delb et al. (2008) found that tinnitus patients with high stress produced greater than normal N1 responses to both attended and unattended ~1-kHz tones [33]. Because neither the Jacobson et al. nor the Delb et al. study tested a tone frequency near the typical tinnitus pitch region (~5 kHz), their positive results suggested an overall attention enhancement in the tinnitus brain but could not address the impact of attention on cortical responses specific to tinnitus frequencies. Paul et al. (2014) [34], on the other hand, addressed this limitation by measuring cortical responses to both 500-Hz and 5-kHz tones but found reduced N1 modulation at both frequencies, opposite to the result from the Jacobson and Delb studies [33]. The reason for this difference was not clear and may be due to the experimental design, in which Paul et al. did not have a selective listening condition with unattended stimuli but instead used passive listening as a reference.

Building upon these previous studies, we used the Jacobson et al. (1996) [32] experimental paradigm, in which subjects selectively attended to one of two interleaved tone streams, including one 5-kHz stream within the tinnitus frequency region and the other 500-Hz stream well below this region. We hypothesized that the difference in the cortical response to the same 500-Hz or 5-kHz tone between the attended and unattended conditions would be greater in tinnitus than control subjects. To further improve the sensitivity of this attention modulation biomarker, we tested the hypothesis using control subjects that were age- and hearing-matched to the tinnitus subjects. To assess the biomarker’s potential clinical utility, we calculated the area under the receiver operating characteristic (ROC) curve, indexing discrimination between tinnitus and control subjects.

## 2. Methods

### 2.1. Subjects

**Figure 1:**
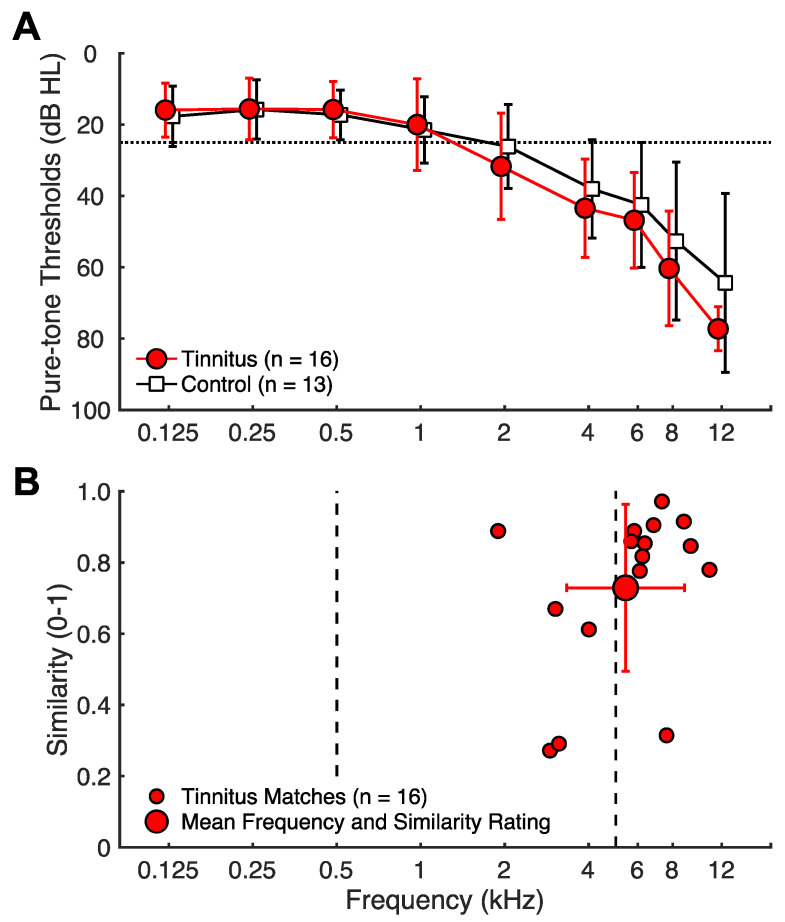
Audiograms and tinnitus matching. (**A**) Audiograms: hearing thresholds in dB SPL as a function of frequency. Tinnitus subjects are depicted by filled red circles and control subjects by open black squares. Error bars indicate 1 SD. (**B**) Tinnitus matching: the *x*-axis indicates the selected tinnitus-matched frequency (kHz), and the *y*-axis indicates the corresponding similarity between the match and actual tinnitus rated on a Visual Analog Scale. Individual subject matches are shown by smaller circles, and the group mean is shown by a larger circle with 1 SD error bars in the frequency and similarity dimensions. Dashed lines indicate test frequencies of 500 and 5 kHz.

A total of 17 tinnitus (5 females) and 14 control (8 females) subjects participated in this study. Inclusion criteria consisted of no self-reported neurological disease or history of significant brain injury, and for tinnitus subjects, having only non-transient, chronic tinnitus for the last 6 or more months. Additionally, one tinnitus subject was excluded due to excessively noisy electroencephalography (EEG). One control subject was excluded due to inability to properly follow task instructions. Thus, 16 tinnitus (5 females) and 13 control (8 females) subjects were included in the final analysis. The tinnitus and control subjects were matched in age (tinnitus: mean = 64 ± 11 SD years; control: mean = 67 ± 13; t = 2.07, *p *= 0.43). They were also matched in hearing thresholds at audiometric frequencies as both had similar age-appropriate sloping hearing loss (F(1,27) = 0.38, *p *= 0.54, see Figure 1A). In addition, there was no significant group difference in the thresholds for the two non-audiometric frequencies (6 kHz: *t*(21) = −0.64, *p *= 0.53; 12 kHz: *t*(17) = −1.55, *p *= 0.14).

All tinnitus subjects completed a comprehensive tinnitus assessment protocol (see Reavis et al., 2012 [35]). They had chronic tinnitus (mean = 21 ± 21 SD years; range = 1 to 58). A total of 12 subjects reported bilateral tinnitus, whereas 4 reported unilateral (3 left ear). In total, 11 subjects reported tonal tinnitus, 3 non-tonal, and 2 mixed. On average, they had moderate tinnitus as assessed by tinnitus loudness (mean = 6 ± 2 on a 0–10 scale), Tinnitus Severity Index (mean = 30 ± 15 on a 0–60 scale), and Tinnitus Handicap Quotient (mean = 29 ± 23 on a 0–100 scale). The subjects matched their tinnitus pitch to a pure tone and rated the match similarity on a 0-to-1 scale (Figure 1B). The average tinnitus pitch was 5425 Hz, and the average similarity rating was 0.73.

All subjects signed their informed consent and received monetary compensation upon completing the experiment. Their consent was provided in accordance with the Code of Ethics of the World Medical Association (Declaration of Helsinki) and approved by the Institutional Review Board of the University of California Irvine.

### 2.2. Stimuli

Two stimuli, 500-Hz and 5-kHz tones, were used in the study. The 500-Hz tone was outside of the tinnitus pitch range, whereas the 5-kHz tone was within the tinnitus range (Norena et al., 2002; Roberts et al., 2006, 2012 [36,37,38], see also Figure 1B). The stimuli had either a standard duration of 60 ms or a longer deviant duration (see Section 2.3). All stimuli were shaped with a 5-ms squared-cosine onset and offset ramps. Two interleaved streams of the 500-Hz or 5-kHz tones were generated, with the inter-stimulus intervals being from 200 to 400 ms, drawn randomly from a uniform distribution with 10-ms steps. The use of this relatively rapid presentation rate minimized attention switching between streams [39,40]. Within each stream, standard tones occurred on 83.3% of presentations, and longer deviant tones occurred on 16.7% of presentations. Tone presentations were pseudo-randomized so that no more than three standard tones of the same frequency occurred consecutively, and no two deviants occurred consecutively. The 500-Hz tone was presented at 65 dB SL, whereas the 5-kHz tone was presented at a level that was loudness-matched by the subject to their 65-dB SL, 500-Hz tone; note, sound levels measured in dB SPL did not differ significantly between groups (mean ± 1 SD control vs. tinnitus at 500 Hz: 87 ± 6 vs. 86 ± 8 dB SPL; 5 kHz: 89 ± 8 vs. 87 ± 10 dB SPL; *p* > 0.05). The stimuli were presented monaurally to the subject. For tinnitus subjects with unilateral tinnitus, the same ear was chosen, while for bilateral tinnitus subjects, the ear with the louder tinnitus was chosen. Ear selection was balanced across control subjects to match the tinnitus group.

Stimuli were generated digitally in MATLAB (The Mathworks, Version 9.0, Natick, MA, USA) and delivered via an external sound card (Creative Labs E-MU 0404 USB digital audio system, Creative Technology Ltd., Singapore, 24-bit, 44.1 kHz) and ER-2 insert earphones (Etymotic Research, Inc., Elk Grove, IL, USA). Transducers were calibrated using a sound level meter with C-frequency weighting in a 2cc artificial ear coupler (Type 2250 Bruel & Kjaer, Nærum, Denmark).

### 2.3. Procedures

**Figure 2:**
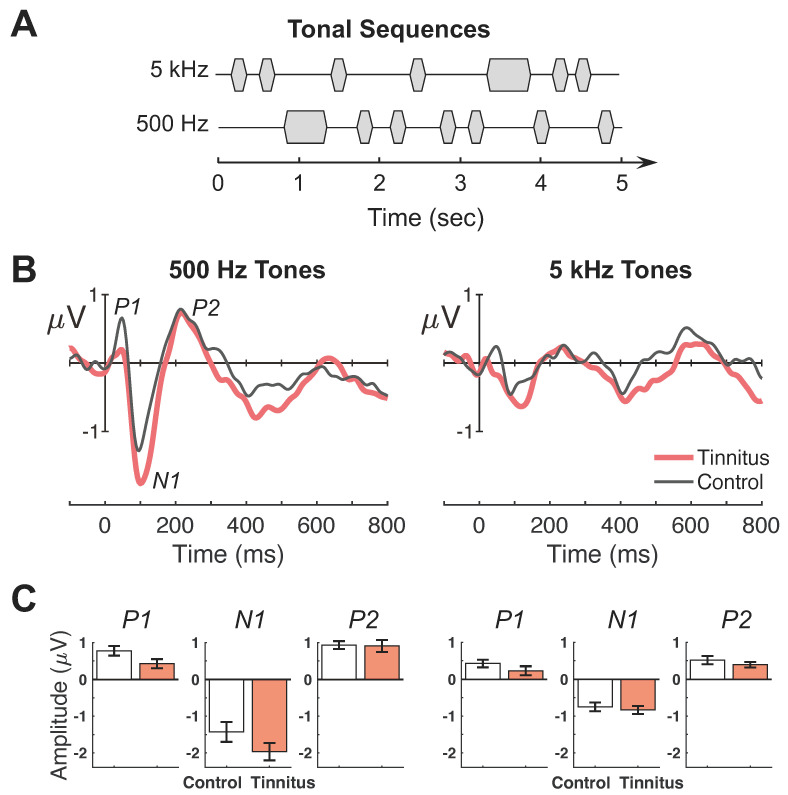
Passive listening evoked potential responses and peak amplitudes. (**A**) Illustrative depiction of the auditory stimuli consisting of tonal sequences at 500 and 5 kHz. Standard tones are the shorter-duration markers and deviant tones are the less frequent long-duration tones. Note: the depiction is not intended to represent exact stimulus parameters. (**B**,**C**) Responses to 500-Hz and 5-kHz tones are shown in the left and right panels, respectively. (**B**) Group-averaged evoked-response waveforms averaged over 64 electrode sites. The *x*-axes indicate time in milliseconds relative to stimulus onset (0 ms) and *y*-axes indicate response amplitudes in microvolts. Each panel contains waveform responses for tinnitus (thick red lines) and control (thin black lines) subjects. (**C**) P1, N1, and P2 peak amplitudes averaged across tinnitus (filled red) and control (open black) subjects. Error bars indicate standard error of the mean.

The experiment consisted of one passive and two attentive listening conditions. For the passive condition, subjects were presented with the two interleaved streams of 500-Hz or 5-kHz tones (Figure 2A) but were instructed to ignore all sounds while reading from a choice of magazines.

**Figure 3:**
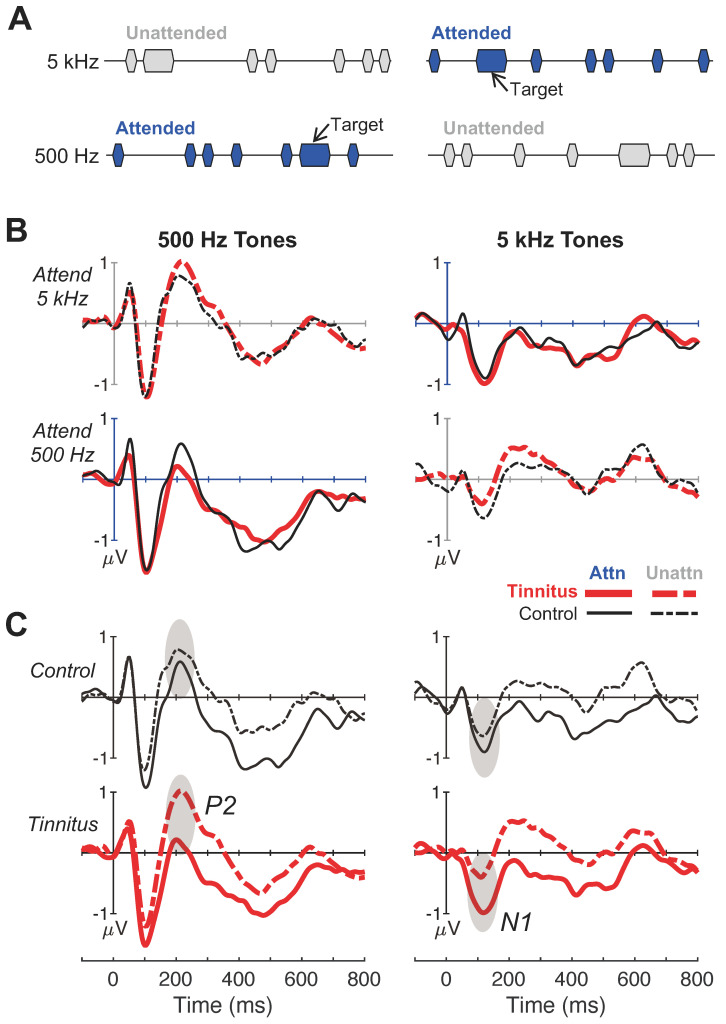
Attentive listening evoked potential responses and peak amplitudes. (**A**) Illustrative depiction of the auditory stimuli and task for the two attentive-listening conditions. Blue markers denote the task instruction to attend either to 500-Hz tones (**left panel**) or 5-kHz (**right panel**) tones, while ignoring the opposite ongoing tone frequency (i.e., gray markers); this procedure produced four attentive-listening response categories: attended—500 Hz, unattended—500 Hz, attended—5 kHz, unattended—5 kHz. (**B**,**C**) Responses to 500-Hz and 5-kHz tones are shown in the left and right panels, respectively. (**B**) Group-averaged evoked-response waveforms averaged over 64 electrode sites. The *x*-axes indicate time in milliseconds relative to stimulus onset (0 ms) and *y*-axes indicate response amplitudes in microvolts. Each panel shows overlapping responses for both subject groups (tinnitus = thick red, control = thin black tinnitus) to either attended (solid lines) and unattended (dashed lines) tones; the attend—5 kHz and attend—500 Hz conditions are shown across top and bottom panels, respectively. (**C**) The same evoked-response waveforms as in (**B**) but replotted to highlight attention modulation of cortical responses to the same stimulus. Each panel shows overlapping responses to attended and unattended tones of the same frequency within each subject group; control and tinnitus are shown across top and bottom panels, respectively.

For the attentive conditions, subjects were instructed to attend only to either the 500-Hz or 5-kHz stream (blue markers, Figure 3A). To orient the subject’s attention toward the correct stream, a train of 10 tones only at the attended frequency was presented at the beginning. Longer-duration deviant tones within the attended stream are called “targets”. The subjects were asked to press a button as quickly and accurately as possible when they detected a target within the attended stream. Performance was measured by a percentage of correct responses and reaction times within a window of 450–1200-ms post-target onset. False alarms were counted as any responses outside of this time window. The target duration was adjusted per subject and frequency to achieve similar performance of 80–90% correct detection. This duration adjustment helped ensure that any potential differences between groups and frequencies were driven by relevant intrinsic neural properties, rather than by task difficulty. Indeed, performance was well balanced with no significant differences by group or stimulus frequency (*p >* 0.5) for both percent correct scores (mean ± 1 SD Control vs. Tinnitus 500 Hz: 86 ± 7 vs. 83 ± 11, 5 kHz: 86 ± 6 vs. 84 ± 10) and false alarms (500 Hz: 1 ± 1 vs. 3 ± 2, 5 kHz: 1 ± 1 vs. 2 ± 2). Reaction times also did not differ significantly between groups (mean ± 1 SD control vs. tinnitus 500 Hz: 721 ± 63 vs. 709 ± 52, 5 kHz: 755 ± 58 vs. 722 ± 58; *p* > 0.05); however, there was a main effect of stimulus frequency whereby reaction times were 23 ms faster for the 500-Hz target than the 5-kHz target (F(1,27) = 4.9, *p *= 0.036).

The experiment always started with a training procedure to familiarize the subjects with the task and determine individual target durations. There were two blocks of formal data collection. The first block started with the passive condition, followed by the two attentive conditions. After a 5–10 min break, the second block started with the attentive conditions, concluding with a second run of the passive condition. The order of the attentive conditions was counter-balanced across subjects. For each condition, an experimental run consisted of 360 tones, including 180 tones for each stream (150 standards, 30 deviants).

### 2.4. Data Acquisition and Analysis

Electroencephalography (EEG) signals were recorded with a Neuroscan SynAmp2 system using Scan 4.5 software and a QuikCap 64-channel cap following the standard 10/20 configuration (Compumedics, Melbourne, VIC, Australia). A vertex reference channel was located between Cz and CPz, and all impedances were monitored to be 10 kΩ or below. Continuous online data were digitalized at 2000 Hz, filtered between DC-500 Hz, and stored for offline analysis. All EEG analysis was conducted using MATLAB (The Mathworks, Version 9.0, Natick, MA, USA). During the EEG recordings, subjects were seated upright facing a computer monitor that displayed the task instruction. During the passive runs, subjects were asked to minimize body movement while reading. In the attentive conditions, subjects looked forward at a fixation cross on the monitor, restricting movements to finger presses on a keyboard.

The EEG data were first down-sampled to 250 Hz, band-passed between 0.1 and 50 Hz (second-order Butterworth, MATLAB filtfilt function), and re-referenced to the average mastoid channels. Channels containing amplifier artifacts were replaced by spline interpolation of the neighboring electrodes. The data were then submitted to independent component analysis using a standalone version of the Infomax ICA algorithm from the EEGLAB toolbox [41]. The components were reviewed to identify those containing activity attributable to blinks or horizontal eye movements. On average, 2.6 (SD = 1.6) components per subject were removed. Finally, the data were transformed back to channel space and screened for excessively noisy trials related to body movements or other transient artifacts. For each channel, a normalized variance was calculated by dividing the variance of each trial by the average variance across trials. A threshold was set for the maximum normed variance, and trials were rejected if this threshold was exceeded on 20% or more electrodes. Thresholds were adjusted such that no more than 10% of trials were rejected per dataset.

Event-related potentials (ERPs) were extracted by 20 Hz lowpass filtering (second-order Butterworth) and averaging across trials. A baseline correction was applied by subtracting the mean voltages from a 200-ms pre-stimulus window from each time point. Only ERPs to standard stimuli were analyzed as deviant ERPs if they contained too few trials to obtain reliable waveforms. To avoid confounding effects of differences in scalp voltage topography for each probe frequency, the analysis focused on an “Average Channel” derived as the grand mean voltage waveform across all the electrodes. All ERP waveforms were analyzed by extracting P1-N1-P2 responses within 30–80, 60–150, and 150–250 ms, respectively.

### 2.5. Statistical Analysis

A two-way mixed analysis of variance (ANOVA) was used to assess significant differences for the between-subjects factor (tinnitus vs. control) and the within-subject factors (500 Hz vs. 5 kHz). Where stated, a one-way ANOVA assessed between-subject differences at individual stimulus frequencies. Significance was assessed at the level of *p <* 0.05 with Bonferroni corrections applied for multiple comparisons across three listening conditions (i.e., passive and two attentive conditions). Effect sizes for significant effects were reported as Eta Squared (η^2^; one-way designs) and Partial Eta Squared (η_p_^2^; two-way designs), with 0.02, 0.13, and 0.26 representing small, medium, and large effects [42].

To test the potential utility of the cortical potentials as a biomarker for tinnitus, two ERP parameters were used to construct the receiver operating characteristic (ROC) curve [43]. The area under the curve (AUC) was calculated, with a value of 0.8 or greater being potentially clinically useful [44]. SPSS was used for the ROC analysis (IBM SPSS Statistics, Version 25.0, Armonk, NY, USA).

## 3. Results

### 3.1. Cortical Responses in Passive Listening

Figure 2B shows average waveforms for the passive condition (left panel = 500 Hz, right = 5 kHz; red lines = tinnitus; black lines = control). All subjects exhibited a typical complex of P1-N1-P2 wave peaks. Figure 2C depicts the individual peak amplitude between the tinnitus and control groups (left panel = 500 Hz, right = 5 kHz). The 500-Hz tone produced larger responses than the 5-kHz tone for all peaks (F(1,25) = 7.93–23.06, *p *= 0.00006–0.009, η_p_^2^ = 0.24–0.48). There was no significant difference in any peaks between the tinnitus and control subjects (F(1,25) = 0.45–3.86, *p *= 0.06–0.51, η_p_^2^ = 0.02–0.13).

### 3.2. Cortical Responses in Attentive Listening

Figure 3B shows average ERP waveforms for the two attentive conditions (left panels = 500 Hz, right = 5 kHz). Similar to the passive condition, the 500-Hz tones elicited larger responses than the 5-kHz tones (F(1,27) = 8.58–28.94, *p *= 0.00001–0.007, η_p_^2^ = 0.24–0.52), and there was no significant difference in any peaks between the tinnitus and control subjects (F(1,27) = 0.01–3.92, *p *= 0.06–0.92, η_p_^2^ = 0.00–0.13).

However, an interesting pattern of results emerged by quantifying cortical responses to the same stimulus between the attended and unattended conditions. Figure 3C replots the same data as in Figure 3B to compare waveforms across attention conditions for the same frequency within subjects. Note that attended responses had a pronounced negative displacement relative to the unattended responses for both frequencies in both groups. In particular, the tinnitus subjects showed greater attention modulation than the control subjects for P2 at 500 Hz and for N1 at 5 kHz (shaded ovals).

**Figure 4:**
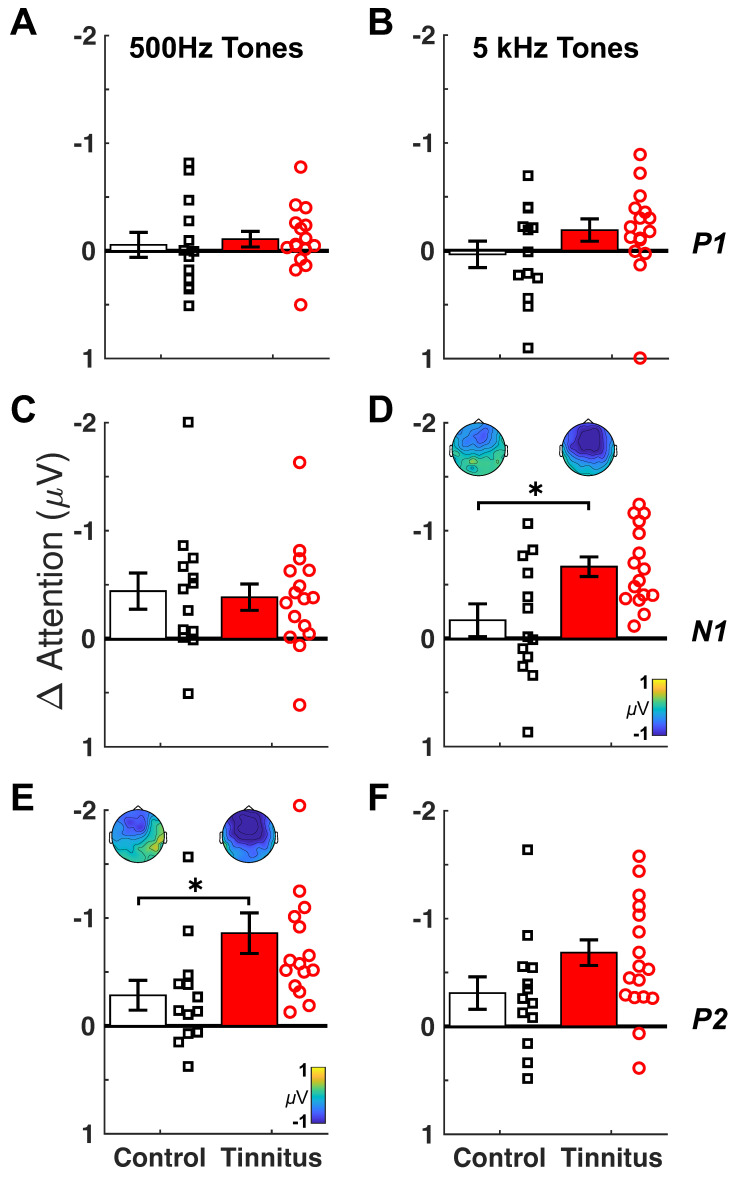
Attentional modulation of the cortical responses: (**A**,**B**) P1, (**C**,**D**) N1, (**E**,**F**) P2. Peak differences between attended and unattended responses are shown for 500 Hz (left panels) and 5 kHz (right panels). Each panel shows group averages and corresponding individual data for control subjects (black, square markers) and tinnitus subjects (red, circle markers). Error bars indicate standard error of the mean. Negative values are inverted in the upward direction. Significant group differences are shown with corresponding scalp voltage topographies averaged across subjects at all electrode sites (* *p* < 0.05).

**Figure 5:**
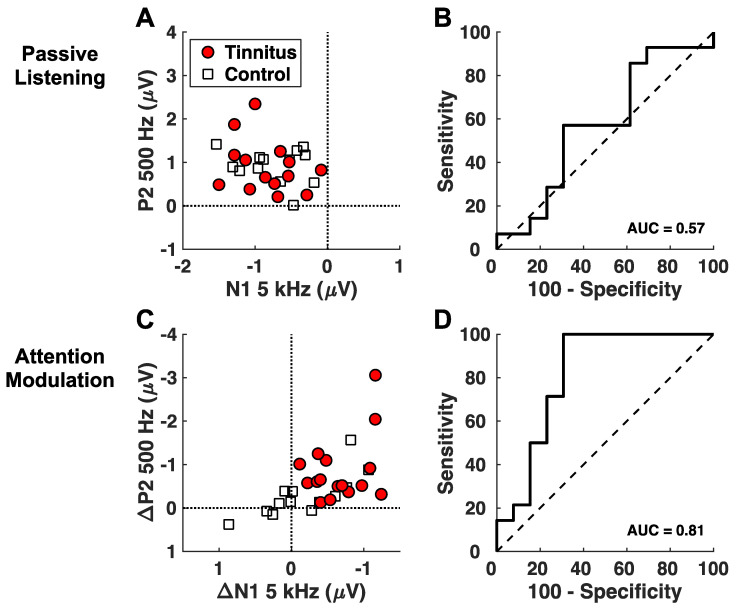
Discrimination analysis. Scatterplots and receiver operating characteristic (ROC) curves for the passive listening (**A**,**B**) and attention modulation (**C**,**D**) variables entered as predictors in separate logistic regression analyses. ROC curves were derived from the predicted probabilities of individual subjects belonging to the tinnitus or control groups obtained in the resulting regression models. Area under the ROC curve (AUC) assessed the overall discrimination ability of each model to correctly classify tinnitus and control subjects using the following interpretation: 0.5 = no discrimination, 0.5–0.7 = poor discrimination, 0.7–0.8 = acceptable discrimination, 0.8–0.9 = excellent discrimination, 0.9–1.0 outstanding discrimination [45].

Figure 4 quantifies the attended–unattended peak difference for 500 Hz (left panels) and 5 kHz (right panels). Three observations were worth noting. First, there was no significant difference in attention-modulated P1 between the tinnitus and control subjects for either 500 Hz (F(1,27) = 0.16, *p *= 0.69, η_p_^2^ = 0.006, Figure 4A) or 5 kHz (F(1,27) = 1.98, *p *= 0.17, η_p_^2^ = 0.07, Figure 4B). Second, while no N1 group difference was present at 500 Hz (F(1,27) = 0.08, *p *= 0.78, η_p_^2^ = 0.003, Figure 4C), the attention-modulated N1 for the 5-kHz tone was 3.9-times greater (95% CI: 2.9 to 5.0) in the tinnitus than control subjects (0.66 vs. 0.17 mV; F(1,27) = 8.54, *p *= 0.007, η_p_^2^ = 0.24, Figure 4D); this was also reflected by a significant group x frequency interaction (two-way ANOVA: F(1,27) = 5.86, *p *= 0.02, η_p_^2^ = 0.18). Lastly, the attention-modulated P2 seemed to show an overall frequency-independent effect. The tinnitus subjects produced 3.0-times greater (95% CI: 1.9 to 4.5) attention-modulated amplitude than the control subjects for the 500-Hz tone (−0.86 vs. −0.28; F(1,27) = 5.56, *p *= 0.03, η_p_^2^ = 0.17, Figure 4E), whereas the 2.2-times greater response at 5 kHz just missed the significance threshold (−0.68 vs. −0.31 F(1,27) = 3.91, *p *= 0.06, η_p_^2^ = 0.13, Figure 4F); indeed there was a significant main effect of tinnitus across frequencies (two-way ANOVA: F(1,27) = 6.31, *p *= 0.018, η_p_^2^ = 0.19). For both significant N1 and P2 measures, the scalp topography showed a more negative response, especially over frontocentral regions, in the tinnitus than the control brain (Figure 4D,E).

Figure 5 shows the results for assessing the attention-modulated responses as a candidate biomarker for tinnitus. A discrimination analysis was applied to the N1 and P2 components. As a basis for comparison, N1 and P2 for the passive condition could hardly differentiate the tinnitus subjects from the control subjects (Figure 5A), which was confirmed by the near chance AUC value (= 0.58, 95% CI 0.35–0.80, *p *= 0.46; Figure 5B). By contrast, the attention-modulated N1 at 5 kHz and P2 at 500 Hz could discriminate between the tinnitus and control subjects with 83.3% sensitivity and 76.9% specificity (AUC = 0.81, 95% CI: 0.64–0.99, *p *= 0.003, Figure 5C,D).

## 4. Discussion

The present study recorded cortical potentials to 500-Hz and 5-kHz tones under passive and attentive listening conditions in groups of age- and hearing-matched tinnitus and control subjects. The 500-Hz was below while the 5-kHz probe was within the tinnitus pitch range. There was no significant group difference in the individual peaks, P1, N2, and P2, of cortical responses to either 500-Hz or 5-kHz tones under any conditions. However, a significant difference emerged when cortical responses were compared between attended and unattended stimuli. Compared with the control, tinnitus enhanced the attention-modulated cortical responses with a 3.9-times greater N1 difference at 5 kHz (large effect size) and a 3.0-times greater P2 difference at 500 Hz (medium effect size).

### 4.1. Comparison with Previous Studies

The present result of enhanced attention modulation in tinnitus was partially consistent with previous studies using a similar experimental paradigm. For example, Jacobson et al. (1996) found that tinnitus subjects had larger N1 responses to attended 500- or 1000-Hz tones in tinnitus than in control subjects (Figure 2 in [32]). The present study found significant group difference in N1 at 5 kHz but not 500 Hz. Delb et al., 2008, also reported enhanced N1 responses to both attended and unattended tones near 1 kHz in a subset of tinnitus subjects with high distress (Figure 3 in [33]). They did not compare the N1 difference between attended and unattended stimuli, nor did they collect any data near the tinnitus pitch (3313–6094 Hz, Table 1 in [33]). The present experimental stimuli were most similar to that of Paul et al. (2014), who collected both transient and steady-state cortical responses to attended or passive 500-Hz and 5-kHz tones [34]. Similar to the present result, they did not find any significant group difference in the passive listening condition, nor a difference in N1 and P2 under any listening conditions. Different from the present study, they found a reduced attention-modulated cortical response to the 5-kHz tone (Figure 5 in [34]). There were two differences between the present study and that of Paul et al. First, we used a longer duration, whereas they used higher-amplitude stimuli as the deviant target. Second, we interleaved the attended stream with an unattended stream, requiring selective listening to only one, whereas they used passive listening to a single-frequency stream as the reference. Although most studies showed greater attentive- than passive-listening effects, the inconsistent results suggest a need to refine the attentional paradigm for using the cortical potentials as a biomarker for tinnitus.

### 4.2. Mechanisms of Attention Modulation in Tinnitus

The present result of the enhanced attention-modulated N1 and P2 may be related to bottom-up and top-down processes in the brain [46]. First, the enhanced attention-modulated N1 response is specific to the 5-kHz tone (Figure 4D), which is close to the tinnitus pitch of 5.4 kHz (Figure 1B). This relatively high-frequency region may have either overt or hidden hearing loss [47], requiring increased central gain or noise [7,8]. This altered bottom-up process induces plastic changes in the tinnitus frequency region of the auditory cortex [36,48,49,50], requiring possibly higher-than-normal attention to modulate the central gain or noise in this tinnitus region to compensate for the reduced input [10,51,52,53,54,55]. In contrast, the bottom-up process at 500 Hz is still intact in tinnitus subjects, producing a normal attention-modulated N1 difference.

Second, the enhanced attention-modulated P2 response at 500 Hz may reflect the altered top-down processing in the tinnitus brain [15,17]. Different from N1, P2 is associated with higher-order sensory and cognitive processing, such as stimulus classification, attention, and memory [56,57,58,59,60,61,62,63]. Chronic tinnitus, as a constant internal signal, increases the cognitive load, requiring more neural resources than the non-tinnitus brain, to perform the same attention task [4,64]. Theoretically, this altered higher-order processing should not be frequency specific, reflecting the increased overall attention or vigilance [65,66,67]. Indeed, we saw a clear trend for the enhanced P2 difference at 5 kHz (Figure 4F). The near-miss to a positive significance test (*p *= 0.06) could be due to relatively smaller cortical responses to the 5-kHz than 500-Hz tone (Figure 2 and Figure 3), the small sample size, or the tinnitus-specific bottom-up attention effect that might mask the overall top-down effect.

The present study also suggests that the enhanced attentional modulation is associated with increased activities in the frontocentral regions of the tinnitus brain (Figure 4D,E). This enlarged cortical activity is consistent with neuroimaging evidence, showing that tinnitus induces changes not only in the auditory cortices but also areas associated with attention and executive function [14,16,22,68,69,70].

### 4.3. Clinical Implications

Currently, tinnitus diagnosis relies on self-reports and urgently needs an objective biomarker. The search for such a biomarker has included various neural measures from auditory brainstem responses (e.g., [6]) to functional and connectivity-based brain imaging [71,72]. However, these biomarkers have not been used widely in clinical diagnosis and practice due to either relatively small neural activities requiring highly sensitive measurement or inconvenient, expensive equipment (e.g., [73]). The present attentional-modulated cortical responses may serve as a biomarker that has not only clinically-meaningful sensitivity and specificity (AUC = 0.81; [45]) but also relatively large neural activity and is easier and cheaper to operate clinically than brain imaging equipment.

The present cortical response paradigm has another advantage because it is an attention-related biomarker. Currently, clinical interventions like cognitive behavioral therapy and perceptual training [74,75,76,77,78] attempt to manipulate attention away from tinnitus to facilitate its habituation. The present attention modulation biomarker can be combined with these approaches for monitoring progress or even developing personalized treatment.

### 4.4. Limitations and Future Directions

Although we controlled for factors of aging, hearing loss, and stimulus salience, the present study is limited by the small sample size, and validation of the proposed attention-related biomarker requires assessment in an independent sample group. The present experimental design also cannot address how the relevant attention-modulated responses might covary with different stimuli and task procedures (e.g., probe frequencies) or subject selections (e.g., young normal-hearing listeners). Lastly, while the results showed a N1 attention effect at the tinnitus-relevant 5-kHz frequency, future approaches might improve upon the biomarker’s tinnitus-specific sensitivity by tailoring stimuli to the subjects’ individual tinnitus attributes (e.g., pitch, bandwidth). 

## 5. Conclusions

The present study designed a novel experimental paradigm to measure cortical responses to interleaved attended and unattended 500-Hz or 5-kHz tones. Compared to age- and hearing-matched controls, tinnitus subjects showed a three-fold greater difference between the attended and unattended responses for N1 at 5 kHz and P2 at 500 Hz. This greater attention modulation for N1 at 5 kHz is interpreted as enhanced attention to the reduced input in the tinnitus frequency region of the auditory cortex, whereas that for P2 at 500 Hz is interpreted as enhanced global attention or vigilance in higher-order brain regions. The present result indicates that the tinnitus brain is more plastic than the age- and hearing-matched control brain. Clinically, the attention-specific cortical measure may serve as a biomarker for not only diagnosing tinnitus but also monitoring its treatment effectiveness.

## Data Availability

The data presented in this study are available on request from the corresponding authors. The data are not publicly available due to privacy restrictions.
